# Vaccination sequence effects on immunological response and tissue bacterial burden in paratuberculosis infection in a rabbit model

**DOI:** 10.1186/s13567-016-0360-y

**Published:** 2016-08-05

**Authors:** Rakel Arrazuria, Elena Molina, Joseba M. Garrido, Valentín Pérez, Ramón A. Juste, Natalia Elguezabal

**Affiliations:** 1 Animal Health Department, NEIKER-Instituto Vasco de Investigación y Desarrollo Agrario, Berreaga, 1, 48160 Derio, Bizkaia Spain; 2 Department of Animal Health, Faculty of Veterinary Medicine, University of León, León, Spain; 3 Department of Agriculture of the Regional Government of the Principality of Asturias, SERIDA, Deva, Asturias Spain

## Abstract

Paratuberculosis (PTB), a chronic granulomatous enteritis produced by *Mycobacterium avium* subspecies *paratuberculosis* (MAP), is considered as one of the diseases with the highest economic impact in the ruminant industry. Vaccination against MAP is recommended during the first months after birth on the basis that protection would be conferred before the first contact with mycobacteria. However, little is known about the therapeutic effect of MAP vaccination in controlled experimental conditions. The current study was designed to evaluate the efficacy of vaccination before and after challenge with MAP in a rabbit infection model. The rabbits were divided into four groups: non-infected control (NIC, *n* = 4), infected control challenged with MAP (IC, *n* = 5), vaccinated and challenged 1 month after with MAP (VSI, *n* = 5) and challenged with MAP and vaccinated 2 months later (IVS, *n* = 5). The results from this study show a quick increase in IFN-γ release upon stimulation with bovine, avian and johnin PPD in animals vaccinated before MAP challenge. All vaccinated animals show an increased humoral response as seen by western blot and ELISA. The final bacteriology index (considering tissue culture and qPCR) shows that the IC group was the most affected. Vaccination after infection (IVS) produced the lowest bacteriology index showing significant differences with the IC group (*p* = 0.034). In conclusion, vaccination against MAP shows positive effects in a rabbit model. However, vaccination after infection shows a slightly stronger protective effect compared to vaccination before infection, suggesting a therapeutic effect. This feature could be applied to previously infected adult animals under field conditions.

## Introduction

Paratuberculosis (PTB) also called Johne’s disease is a chronic granulomatous enteritis produced by *Mycobacterium avium* subspecies *paratuberculosis* (MAP) that mostly affects ruminants. PTB considered as one of the diseases with the highest economic impact in the ruminant industry can affect many ruminant and non-ruminant wildlife species, as well [1, 2]. In addition numerous studies have suggested that MAP could play a role in human Crohn’s Disease [3–5].

PTB is transmitted predominantly through the fecal-oral route. Infected animals in the subclinical stage shed MAP in the environment intermittently, making its control difficult. Current control programs in livestock include test and cull strategies whose implementation alone is not sufficient to control the disease making vaccination the best intervention strategy [6, 7]. Vaccination against MAP reduces the incidence of PTB within herds and the severity of disease in individual animals. In general, it reduces production loss, mortality and histopathological lesions [8, 9] and it diminishes the number of clinical cases in cattle, reducing bacterial shedding in feces contributing to longer productive life and improvement in milk production [10–12]. In small ruminants, vaccination has been successfully used as a part of the control programs in sheep in Iceland [13] and Australia [14] and in goats in Norway [15] and Spain [16] allowing a sharper decline in PTB prevalence. However, the fact that vaccination does not provide complete protection from infection is also widely accepted [17, 18].

Vaccination against MAP is recommended during the first months after birth on the basis that protection would be conferred before the first contact with the mycobacteria [6, 15, 19]. Nevertheless, due to the chronic nature of MAP infection when clinical signs are detected vaccination of the whole flock independent of age, including adult animals can be an option [12]. In fact, studies involving vaccination after MAP infection have shown reduction of MAP burden in sheep vaccinated with Gudair^®^ [20] or with a live attenuated vaccine [21]. Although the mechanism of this therapeutic effect is unknown it can be assumed that whole herd vaccination might contribute to a reduction in the level of environmental contamination with MAP allowing faster progress in PTB control.

The therapeutic effect of vaccines could be very useful controlling the spread of infectious diseases in which the number of individuals in a latent or subclinical stage of infection is high. A recently published systematic review has reported that some vaccines against tuberculosis currently under study have a therapeutic effect even in latent infection [22]. When it comes to PTB, vaccination effects after MAP exposure have been understudied in experimental conditions, probably because of the lengthy incubation period of PTB in ruminants. In this sense, rabbits which are naturally susceptible to infection with MAP [23–25] and have shown to be useful short term models in experimental conditions as well [26], may be suitable to study the therapeutic effect of PTB vaccination.

The aim of this study, therefore, was to evaluate the effect of vaccination with a heat inactivated vaccine previous to and after challenge with MAP in a rabbit infection model resembling the subclinical stage of PTB, focusing on changes in bacterial load and both cellular and humoral responses.

## Materials and methods

### Experimental design

**Figure 1:**
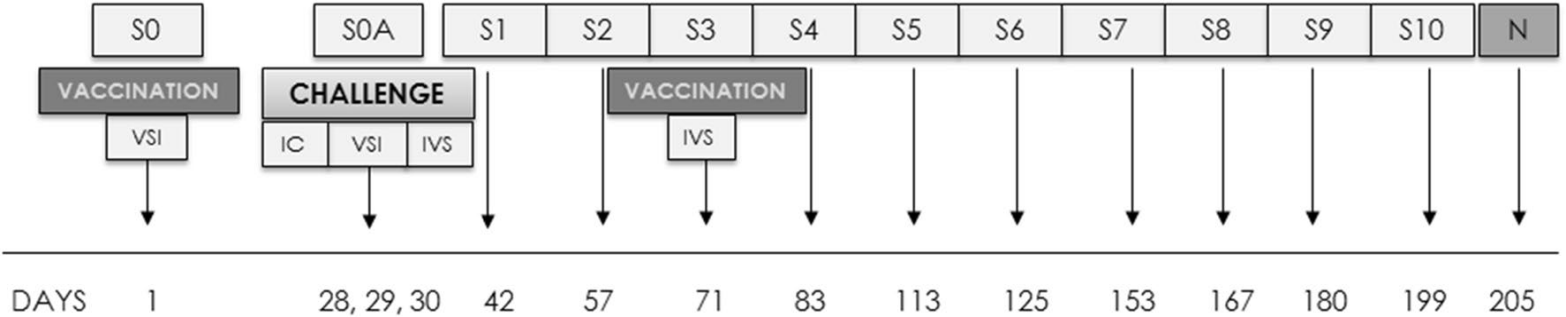
**Experimental design diagram.** S: sampling, N: necropsy, IC: infected control, VSI: vaccination before infection, IVS: vaccination after infection.

New Zealand white female rabbits (*n* = 19) were purchased from official dealers and arrived at the NEIKER animal facilities at the age of 6 weeks 1.5 kg weight to follow a 2 week adaptation period. The most important aspects of the experimental design are represented in Figure 1. The rabbits were divided into four groups: non-infected control (NIC; *n* = 4), infected control (IC; *n* = 5), infected and then vaccinated with Silirum^®^ (IVS; *n* = 5) and vaccinated with Silirum^®^ prior to infection (VSI; *n* = 5). Silirum^®^ is a heat-inactivated vaccine containing 2.5 mg/mL of MAP strain 316F culture combined with an immunological adjuvant consisting of highly refined mineral oil.

At day 1 of experimentation, animals were sedated with azepromazine maleate intramuscularly at 2 mg/kg of weight and tattooed in the inner part of one of the ears for easier identification during the experiment. Also, on this day, the VSI group rabbits were vaccinated subcutaneously with 1 mL of Silirum^®^. On three consecutive days (days 28, 29 and 30) all animals except the ones belonging to the NIC group were challenged orally with 10^9^ CFU of MAP strain K10 obtained as described previously [26]. Rabbits of the IVS group were vaccinated with Silirum^®^ 71 days after the beginning of the experiment that is, 41 days after challenge.

Animals were monitored by recording weight and blood prior to vaccination (baseline S0 samples) and after challenge twice a month (S1–S10). The fecal consistency was observed in all samplings. Feces were collected after challenge (S0A) and freshly processed. Blood samples were divided for IFN-γ release quantification and for serological testing. In this case, blood was centrifuged at 1500* g* for 10 min to obtain plasma which was stored at −20 °C until ELISA or Western blot (WB) analyses were performed.

The endpoint of the experiment was set at day 205 and all animals were euthanized administering pentobarbital intracardially after deep sedation with xilazine and ketamine. Then a complete necropsy focusing on a gross pathology in the digestive tract was performed and tissue samples were collected and stored at −20 °C. Handling procedures, sampling frequency and euthanasia procedure were designed to produce minimum stress on subjects following European, National and Regional Law and Ethics Committee regulations. The experiment was reviewed and approved by NEIKER’s Animal Care and Use Committee and by the Agriculture Department (PARAMOD-6278-BFA).

### Tissue MAP culture

For culture, 2 g of collected tissues were processed. Tonsils, spleen, liver, muscle and mesenteric lymph node were spliced in tiny pieces and weighed, whereas, vermiform appendix, sacculus rotundus, ileum and jejunum were scraped for mucosa and weighed and samples were processed for HEYM [27] as described in Arrazuria et al. [26]. For culture on 7H9 OADC [28] supplemented with MJ penicillin, anfotericin and cloramphenicol, the same decontaminated suspension used for HEYM culture was centrifuged at 2885 *g* during 10 min. Supernatant was discarded and a wash with sterile water was performed. After a new centrifugation step in the same conditions the pellet was suspended in 2 mL of water and four drops/tube were seeded. All seeded tubes were incubated at 37 ± 1 °C and checked for MAP growth at 8, 12, 16 and 20 weeks.

### Fecal and tissue DNA extraction

Feces and tissue DNA was extracted with DNA Extract-VK (Vacunek S.L, Derio, Spain) according to the manufacturer’s instructions with slight modifications that included an initial hydrating step where 1 g of feces was hydrated with 7 mL of sterile water overnight. The following day, the mixture was vortexed to achieve a homogenous solution and let still for 10–15 min. Then 500 µL of supernatant were pipetted into a new vial and centrifuged at 11 000* g* for 5 min. The supernatant was discarded and the pellet was suspended in 250 µL of sterile water. From this step on, the manufacturer’s instructions were followed.

Collected tissues (tonsils, spleen, liver, muscle, mesenteric lymph node, vermiform appendix, sacculus rotundus, ileum and jejunum) and cecal content stored at −20 °C were thawed overnight at 4 °C and brief modifications were performed as previously described [26].

In both cases extracted DNA was stored at −20 °C until PCR were performed.

### MAP PCR. Detection and quantification

For initial screening purposes a real time multiplex PCR detecting IS900 and ISMap02 DNA sequences of MAP was performed with 350 ng/μL as described by Sevilla et al. [29].

PCR results were analyzed using the 7500 System SDS software v. 1.4 (Applied Biosystems, Spain). Threshold cycle (C_*T*_) and baseline were automatically determined by the software and verified by visual examination of the threshold line in amplification plots. C_*T*_ values equal or below 38 for both IS900 and ISMap02 probes were considered positive, C_*T*_ values over 38 for both targets probe and under 38 for IAC probe were considered negative.

For a quantitative assessment of MAP levels in tissues, this is genomic equivalents (GE), qPCR was done employing ParaTB-Kuanti-VK (Vacunek S.L, Derio, Spain), and the manufacturer’s instructions were followed. Briefly, 10 µL of DNA were added to 15 µL of master mix in 96 well plates. Triplicates of dilutions of positive control standards were run in parallel to obtain data points for standard curves. Amplification and real time measurement were performed in a 7500 Real Time PCR System (Applied Biosystems, Spain). The results were analysed with the ABI Prism software version 7500 SDS software v 1.4.

### Interferon gamma release assay (IGRA)

To explore the cellular immune response, interferon gamma release quantification was carried out after stimulation of whole blood with bovine, avian and johnin purified protein derivative (PPD) (CZ Veterinaria, Porriño, Spain). Heparinized blood samples collected at S0, S1, S3 and S10 were assayed. Stimulation of whole blood was performed within 8 h of collection. A 25 000 UI/mL concentrations of bovine and avian PPD and 26 446 UI/mL of johnin PPD were used.

Briefly, 35 µL of different PPD (10 µg/mL) or sterile PBS 0.01 M pH 7.2 used as a negative control were added to 500 µL of whole blood from each animal. All samples were mixed slowly and incubated at 37 °C and 5–7% CO_2_ for 16–24 h. Plasma was separated by centrifugation at 500 *g* for 10 min and frozen at −20 °C until testing. The IFN-γ production was measured using a commercial kit (Cusabio-Biotech^®^, China) according to the manufacturer’s instructions.

The optical density was determined at 450 and 570 nm to correct for optical imperfections in the plate, using an automated ELISA plate reader (Multiskan EX^®^, Thermo Lab Systems, Finland). The standard curve was performed to calculate the IFN-γ concentration of each sample. The final IFN-γ concentration was obtained by subtracting the values of the negative control (sterile PBS) from each tested PPD in order to reduce the background of non-specific IFN-γ release.

### PPA-3 enzyme-linked immunosorbent assay (ELISA)

Homemade indirect ELISA was performed using PTB protoplasmatic antigen 3 (PPA-3) (Allied Lab, Fayette, Missouri, USA). Plasma samples were adsorbed with *Mycobacterium phlei* in saline solution (5 g/L) at equal concentrations to reduce cross-reactivity. Checkerboard titration was performed to determine the optimal concentrations of plasma (1:200) and conjugate (0.15 µg/mL) (unpublished data).

Microtiter plates were coated with 0.04 mg/mL of PPA-3 diluted in 0.5% sodium carbonate buffer (pH 9.6). A total of 100 µL of antigen dilution was added to each well and it was incubated at 4 °C overnight. After coating, the wells were washed once with 0.05% Tween 80 in 0.85% sodium chloride (Wash solution).

The plasma samples were diluted 1:200 in 0.05% Tween 80 in PBS (PBS-T) and were assayed (100 µL/well). After 2 h of incubation at RT in a humid chamber, plates were washed three times with 300 µL/well of wash solution. A volume of 100 µL of recombinant protein G peroxidase (0.025 µg/mL, Sigma-Aldrich, Spain) in PBS-T was added to each well and the plates were incubated at RT for 2 h. The plates were washed again three times with 300 µL/well of wash solution and then 100 µL/well of peroxidase substrate (0.01% ABTS, Sigma Aldrich, Spain) were added. The plates were incubated at RT for 30 min in darkness. The reaction was stopped by the addition of 100 µL/well of 2% hydrofluoric acid. The absorbance was measured at 405 and 450 nm using an automated ELISA plate reader (Multiskan EX^®^, Thermo Lab Systems, Finland).

The reading obtained at 450 nm was subtracted from the reading of 405 nm to reduce optical imperfections in the plate. The results are expressed as a relative absorbance index calculated by dividing the mean absorbance of the sample by the mean absorbance of a negative sample. Optical density (OD) values were normalized across plates using the following calculation: Absorbance index = (Mean sample OD) × (Mean OD of all positive control plates/Mean OD of the positive control plate).

### Sds-page

K10 strain proteins were obtained and separated by SDS-PAGE 12%. For protein extraction 50 mL (approximately 1.5 mL of cell pellet) of MAP K10 strain culture at log phase was centrifuged at 3000 *g* 10 min. The supernatant was discarded and the pellet was washed three times with PBS 0.01 M. The pellet was resuspended in 300 µL of complete extraction buffer containing 15 µL of DTT (dithiothreitol), 12 µL of IP25 (Protease inhibitor 25x) and 273 µL of extraction buffer. The extraction buffer was composed of Tris 0.04 M, Urea 8 M and Chaps 0.01 M in Ready Prep proteomics Grade Water (Bio-Rad Laboratories, Spain). This suspension was added to tubes with 300 mg of glass beads and homogenized at 30 Hz during 10 min on TissueLyser II (Qiagen, Spain). Following homogenization, centrifugation at 15 000* g* during 15 min was performed and the supernatant was transferred to a new vial. The absorbance was measured at 280 nm in a NanoDrop^®^ ND-1000 Spectrophotometer (Thermo scientific, Spain).

For protein separation, 200 µg of K10 strain proteins were loaded in 12% acrylamide gels, and separation by electrophoresis was performed at 150 V. Precision Plus Protein™ Dual Color Standard (Bio-Rad Laboratories, Spain) was loaded in one lane to monitor electrophoretic separation, transfer efficiency as well as molecular weight size.

### Protein transfer

The membrane protein transfer was performed according to the guidelines outlined by Towbin [30]. Briefly, proteins were transferred to polyvinylidene fluoride (PVDF) microporous membrane, Immobilon^®^ (Millipore Ibérica, Madrid, Spain) by Trans-Blot^®^ Semi-Dry electrophoretic transfer cell (Bio-Rad Laboratories, Spain) at 15 V.

### Western blot (WB)

The membrane was cut in 0.5 cm wide strips that were blocked with 0.1 M Tris-Buffered Saline (TBS) supplemented with 5% nonfat dry milk (TBS-M) overnight at 4 °C.

Plasmas were diluted 1:50 in TBS-M prior to incubation with the membrane for 1 h at 37 °C with slow agitation. After four five minute washes with TBS, membranes were incubated with recombinant protein G peroxidase (Sigma-Aldrich, Spain) 1:8000 in TBS-M for 1 h at 37 °C. The membrane was washed again four times with TBS and enhanced chemiluminescence (ECL) was added. The immunoreactive bands were visualized by autoradiography. After scanning the autoradiography, reactivities were measured with Image J software.

### Statistical analysis

Significance of the differences among groups for all variables weight, fecal PCR, MAP tissue PCR, IFN-γ release increase index, ELISA index, western blot reactivity, bacteriology index were assessed using analysis of variance (ANOVA). Some variables were based on summary measures obtained as follows; weight gain: the difference between S10 and S0 weight, total MAP in tissues: the sum of MAP GE/g in all examined tissues, ELISA index: the difference between S10 and S0 index, Western blot reactivity: the sum of the total reactivity per area for each protein measured with Image J. Non- normally distributed data were subjected to natural log(Ln) transformation prior to statistical analyses. When ANOVA showed significant differences, Tukey’s post hoc testing was used to make paired comparisons. Pearson’s correlation test was applied to assess the association between ELISA and total western blot reactivity.

IFN-γ response was expressed for each sampling time 1, 3 and 10 (S1, S3, S10) after subtracting the start point sampling (S0). In order to estimate the contribution of vaccination to IFN-γ release, an index was calculated, where mean values from non-vaccinated infected animals were subtracted from the value of each vaccinated and infected animal and divided by the value of each vaccinated and infected animal. Afterwards, ANOVA was performed. The percentage of these indexes may be assumed to be equivalent to the response conferred by vaccination.

In order to take into account both bacteriological outcomes, the results of both post-mortem bacteriological techniques (qPCR of tissues and tissue culture) were used to generate what was termed as the bacteriology index. For this purpose, the number of positive qPCR tissues and culture were placed on the same scale by dividing each category by the maximum value within the category. The sum of the two resulting values for each technique was the final bacteriology index for each animal.

All statistical analyses were performed using R statistical software (3.1.0) and differences among groups for all variables were stated at *p* < 0.05.

## Results

### In vivo follow up

All animals maintained a healthy status until the experiment endpoint except for one animal belonging to the VSI group. This animal was euthanized prematurely at 153 days after the beginning of the experiment due to an unrelated pathology.

Challenge was achieved in all animals as shown by equal shedding levels in all animals from the third day challenge fecal sampling (*p* = 0.373). No episodes of diarrhea were observed throughout the experiment. However, fecal consistency did vary from hard to soft pellets occasionally in some animals.

Animals maintained normal food intake and only minimal weight loss was observed in some occasions in individuals that eventually recovered from the losses. Significant differences in weight gain among groups were not observed (*p* = 0.302).

### Cellular immune response: interferon gamma release assay

As expected, animals from the NIC group did not show an increase of IFN**-**γ levels with any of the studied PPD and in the IC group, slightly higher IFN**-**γ levels were observed in the first sampling after challenge with all tested PPD (data not shown). In order, to measure the effect of vaccination on the cellular immune response the percentage of IFN**-**γ levels due to vaccination were calculated and represented on Table 1. These percentages were superior for the VSI group compared to the IVS group upon stimulation with all PPD at all time points showing significant differences at S10 upon stimulation with johnin (*p* = 0.0019).

**Table 1:** **Percentage of IFN-γ release index upon stimulation with PPD due to vaccination**

	PPD	VSI^a^	IVS^b^	*p* value
S1	AV	54.56	0.00	0.172
	BO	60.97	0.00	0.139
	JO	64.66	0.00	0.296
S3	AV	94.21	62.68	0.969
	BO	0.00	1.27	0.111
	JO	62.09	54.59	0.925
S10	AV	100.00	100.00	0.404
	BO	100.00	100.00	0.431
	JO	33.21	0.00	0.0019^*^

AV: Avian PPD, BO: bovine PPD, JO: johnin PPD, VSI: vaccinated before infection, IVS: vaccinated after infection, S1: IFN-γ release index first sampling. S3: IFN-γ release index third sampling, S10: IFN-γ release index tenth sampling.

* *p* < 0.05.

^a^Index percentage in relation to the infected control group (VSI-IC)/VSI.

^b^Index percentage in relation to the infected control group (IVS-IC)/IVS.

### Humoral immune response

**Figure 2:**
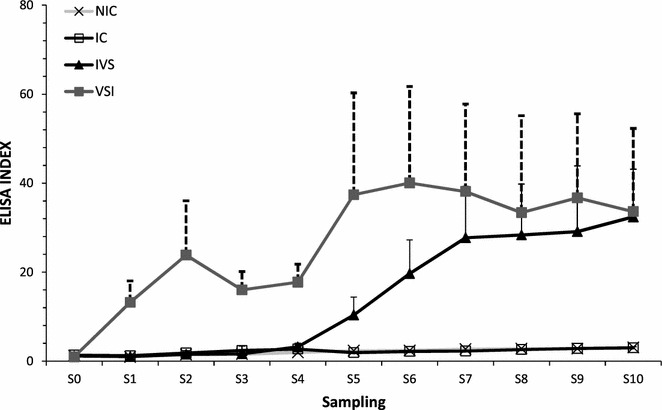
**Evolution of mean ELISA index value per group.** NIC: non infected control, IC: infected control, IVS: vaccination after infection, VSI: vaccination before infection. Error bars represent standard error of the mean.

The antibody production against MAP was assessed by PPA3 ELISA and WB. The results of antibody production expressed as the means of the ELISA Index values of all study groups during the experiment are represented in Figure 2. Vaccination with Silirum^®^ produced an increase in humoral immune response prior to challenge (VSI vs IC; *p* = 0.005) and also after challenge (IVS vs IC; *p* = 0.001). However, infection alone did not raise humoral response, at least during the experimental period (NIC vs CI; *p* = 0.999). Vaccination prior to challenge produced slightly higher levels of antibodies compared to vaccination after challenge; however, at the end of the experiment the level of antibodies was similar in both vaccination schemes (Figure 2).

**Figure 3:**
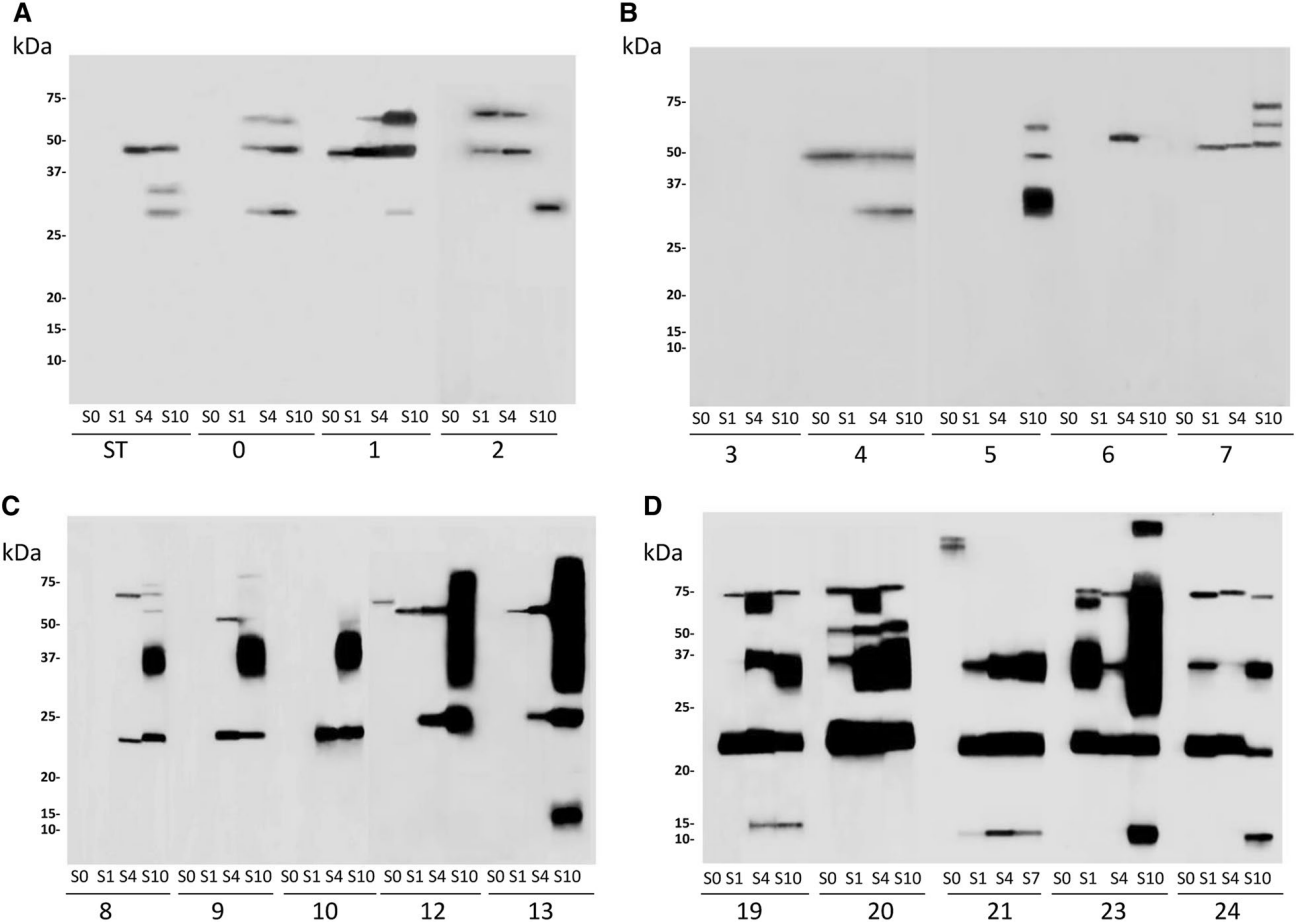
**Western Blot of serum of individual animals showing reactivity to MAP K10 extracted proteins.** **A** Non-infected control (NIC),** B** Infected control (IC),** C** Vaccinated after infection (IVS), and** D** Vaccinated before infection (VSI). Strips were incubated with serum dilution 1:50. Recombinant protein G peroxidase was used at dilution 1:8000. Blots were developed by chemiluminescent detection and visualized by autoradiography. Samplings (S0, S1, S4 and S10) of each animal are represented.

WB results are shown in Figure 3. We did not find any differences in humoral response against MAP proteins between infected and non-infected animals in both assays. The reactivity per area which was measured attending to the protein weight did show differences when vaccinated animals were compared with infected ones, as well as between both vaccinated groups. Humoral response against ~22 kDa protein was significantly higher in both vaccinated groups, vaccinated after challenge (IVS vs IC, *p* = 0.007) and vaccinated before challenge (VSI vs IC, *p* < 0.0001). The VSI group also had higher antibody levels against ~22 kDa than the IVS group (*p* < 0.0001). Animals vaccinated before challenge (VSI) had higher levels of antibodies against ~35 kDa proteins than those of the infected group (VSI vs IC, *p* < 0.0001) or those vaccinated after challenge (VSI vs IVS, *p* < 0.0001). Vaccinated animals after infection (IVS) showed higher reactivity against ~37 kDa protein than IC animals (*p* = 0.008). Regarding humoral response against ~75 kDa proteins the animals vaccinated before infection (VSI) had higher values than IC animals (*p* = 0.030) and the IVS group animals (*p* = 0.014).

The total reactivity per group in WB had a strong positive correlation with the ELISA assay (r = 0.993; *p* = 0.006).

### Post mortem findings

A summary of postmortem findings is shown in Table 2. At necropsy, some infected animals presented gross pathology consisting of increased vascularity and pale-white reactive spots in the sacculus rotundus and vermiform appendix as well as wall thickening in the ileum and jejunum compatible with PTB.

**Table 2:** **Summary of post mortem findings**

Group	Animal	Gross pathology^a^			Tissue qPCR GE/g			Tissue culture			
		SR	VA	MUC	SR	VA	MUC	SR	VA	ICV.IL	MLN
	ST	–	–	–	–	–	–	–	–	–	–
NIC	0	–	–	–	–	–	–	–	–	–	–
	1	–	–	–	–	–	–	–	–	–	–
	2	–	–	–	–	–	–	–	–	–	–
	3	++	++	+	246	42.9	86.7	+	+	+	+
IC	4	+	++	+	–	–	5.13	+	+	–	–
	5	+	+	–	620	–	–	–	–	–	–
	6	+	+	+	–	37.8	–	+	+	–	–
	7	+	+	–	200	105	6.27	+	–	–	–
	8	–	–	–	–	–	–	–	–	–	–
IVS	9	–	++	–	–	–	–	–	–	–	–
	10	–	–	+	–	–	1130	–	–	–	–
	12	–	–	–	–	–	–	–	–	–	–
	13	–	–	–	1690	38.4	–	–	–	–	–
	19	–	–	–	–	258	2.45	+	–	–	–
VSI	20	–	–	–	NA	–	7.16	–	–	–	–
	23	–	–	+	–	–	–	–	+	–	–
	24	–	–	–	–	–	–	–	–	–	–

NIC: non infected control, IC: infected control, IVS: infected before vaccination, VSI: vaccinated before infection, SR: sacculus rotundus, VA: vermiform appendix, MUC: ileum and jejunum mucosa, ICV.IL: ileocecal valve and Ileum, MLN: mesenteric lymph node, NA: not analyzed.

^a^Gross pathology consisting of the following: pale white reactive spots and severe wall thickening (++) and increased vascularity and mild wall thickening (+).

MAP was isolated from the sacculus rotundus, vermiform appendix, ileum and/or mesenteric lymph node in 6 of the 14 infected animals. In the IC group, MAP was isolated from at least one tissue in 80% of the animals. Vaccination decreased the bacterial burden in tissues. MAP was not isolated from any tissue of animals belonging to the IVS group whereas two animals of the VSI group presented one MAP positive tissue culture each.

If we attend to bacterial load measured by qPCR, we detected MAP DNA in the sacculus rotundus, vermiform appendix and mucosa. In the IC group, all animals had at least one tissue positive for qPCR with a mean of 150 ± 87 GE/g of MAP. Vaccination decreased the number of positive tissues for MAP qPCR. MAP DNA was detected in at least one tissue in 40% of the animals from the IVS group while 50% of the animals of the VSI group presented positive results to qPCR. However, bacterial load in the IVS group was higher than in the VSI group (953 ± 375 GE/g of MAP, 94 ± 71 GE/g of MAP respectively) although significant differences were not observed among the vaccinated groups.

**Figure 4:**
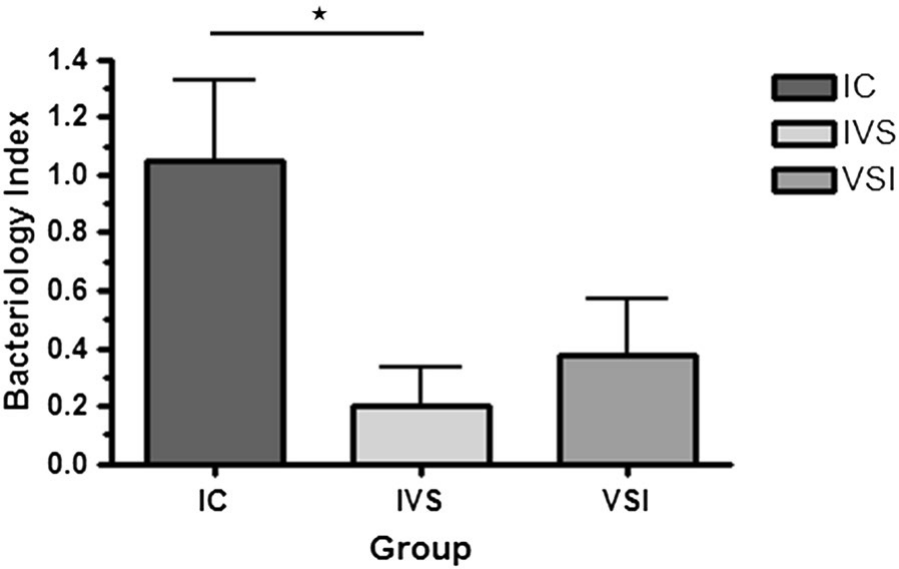
**Bacteriology index per group.** Bacteriology index was calculated based on the number of MAP qPCR positive tissues and the number of MAP culture positive tissues. IC: infected control, IVS: vaccinated after infection, VSI: vaccinated before infection. Error bars represent standard error of the mean. Significant differences were observed between the IC and IVS group (*p* = 0.034).

The bacteriology index considering the number of positive tissues for qPCR and the number of tissues in which MAP has been isolated is represented in Figure 4. Animals of the IC group had the highest values followed by animals vaccinated before infection (VSI). Vaccination after infection (IVS) produced the lowest bacteriology index showing significant differences with the IC group (*p* = 0.034).

## Discussion

The present study attempts to characterize the effect of vaccination before and after MAP challenge in a rabbit infection model. To our knowledge this is the first time that vaccination against PTB has been evaluated in a rabbit model, showing an increased immune response and reducing bacterial burden in tissues.

All challenged animals had equal chances of being infected due to the absence of significant differences in shedding levels in the fecal sampling immediately after challenge. As in previous studies carried out by our group with this short term model, clinical signs were not observed representing the subclinical stage of infection [26]. In order to observe clinical symptoms the experiment should have gone on for a longer period of time: 5–8 months for diarrhea [31, 32] and up to 2 years for emaciation [33].

PTB whole cell vaccine studies have demonstrated the induction of both cellular and humoral immune response in calves [34–37], sheep [38, 39] and goats [40]. For this reason we have attempted to evaluate both: cellular by IFN**-**γ release and humoral by ELISA and Western blot. Animals vaccinated before infection (VSI) had a temporary increase in IFN**-**γ response (at S1 or 42 days after the beginning of the experiment) although significant differences were not observed, probably due to the high variability between animals (non-reactors, low-reactors and high reactors) and in turn by the small sample number. These results are supported by previous studies in which vaccination with a whole cell vaccine increased IFN**-**γ and IL-10 release, in a murine model [41]. In addition, in this group (VSI), significant differences between IFN**-**γ release levels upon stimulation with bovine and avian PPD were not observed as documented in previous studies [34, 42, 43].

The effect of vaccination after challenge in the cellular immune response is not clear, at least on the short term basis. The IVS group was vaccinated at S3 (71 days of the start point of the experiment) and IFN**-**γ response was measured at S10 (128 days after vaccination). In this period of time IFN**-**γ response could have increased temporarily and decreased shortly afterwards, and we would not have detected these oscillations at S10. Also, the effect of vaccination itself prior to challenge was missed since a proper sampling was not performed between vaccination and challenge in the VSI group. For this reason, calculating the index in relation to infection was the only way to estimate the contribution of vaccination on IFN**-**γ release at each sampling time point. The VSI group presented higher percentages of IFN**-**γ index in comparison to the IVS group meaning that on a long term basis vaccination prior to infection may be more effective in maintaining a cellular immune response. In any case, differences between both groups were only significant for johnin PPD at the final sampling point, so we cannot state if there was any difference in the specificity of cellular immune response related with the sequence of vaccination.

Since MAP is an intracellular organism, a Th1 response and in particular IFN**-**γ secretion has been thought to be suggestive of protection in PTB, similar to other mycobacterial infections [44]. However, studies reporting high IFN**-**γ levels in vaccinated animals with high bacterial burden and severe lesions [34–37] point out that IFN**-**γ may be a response to MAP antigens in circulating cells more than a correlate of protection. Recently humoral immune responses have shown to play key roles in protection in PTB [45] and against *M. tuberculosis* infection [46, 47]. Therefore, the increased level of antibodies in both vaccinated animal groups observed in our study could be in part responsible for the lower bacterial burden detected in the analyzed tissues. On the contrary, we did not observe an increased humoral response against MAP in the IC group probably because of the subclinical stage of the disease. In these animals the low antibody titers that they could be harboring may be forming immune complexes not detectable by ELISA or WB. In agreement, previous studies have shown low antibody titers during the first 2 years of infection, in naturally MAP infected cows [48].

In the present study, WB analysis was performed to better characterize host immune responses to vaccination and to identify potential markers that might be useful in differentiating MAP vaccinated and MAP infected animals. Unfortunately the absence of humoral immunity in the IC group during the study period impeded prediction of these hypothetical protein markers.

Although ELISA measures antibody levels against the PPA3 antigen and WB shows reactivity against all MAP extracted proteins, both techniques showed a strong correlation when total reactivity in WB was compared to ELISA index values. At the individual protein level, WB analysis showed some differences in humoral response among studied groups. For example, animals vaccinated before challenge had reactivity against ~12 kDa proteins which was absent in animals vaccinated after challenge. Animals vaccinated before challenge had higher reactivity against 22, 35 and 75 kDa proteins compared to all animal groups. These changes were probably related to the intensity of humoral response, and to the fact that vaccination in the VSI group has had more time to develop. A previously characterized MAP lipoprotein of approximately 22 kDa (P22) has demonstrated the ability to induce humoral and cellular responses being detected in vaccinated sheep and in clinically and subclinically infected cows [49]. In our study, both vaccinated groups had reactivity against a ~22 kDa protein absent in infected animals which may suggest that in our subclinical experimental model the ability to stimulate the humoral response by infection may be more limited than in cows in field conditions.

Vaccination has been reported to be the best currently available tool to control PTB in field conditions in recent reviews [6, 50]. We have noted that both groups of vaccinated animals harbor fewer bacteria in tissues than non-vaccinated animals. However, other vaccination studies performed in a murine model have shown poor protection using the whole cell vaccine Silirum^®^ [51], suggesting that the rabbit model could better emulate the effect of vaccination previously observed in ruminants.

Attending to the bacteriology index, we observed that vaccination after infection was more effective than vaccination before infection. Vaccination in older animals has been reported to induce better immune response [39], ameliorate the clinical disease [52, 53] improve milk production [10] and longevity [11, 54]. Although the classic recommendation to vaccinate animals before being infected has proven to be an effective approach in acute diseases, it might not be an effective strategy in chronic diseases. The goal of vaccination in acute diseases is to mimic natural infection and increase the specific immune response in order to enhance immunity and avoid the disease. However, the acquired immune response might be not sufficient to clear the infection in chronic diseases [55]. Furthermore, an increased immunity produced by vaccination in the clinical phase of the disease could have a beneficial effect and it could be used as a short term individual strategy to control the disease in high valued animals when vaccination of the whole flock is restricted.

In the present study we used a rabbit model, showing positive effects of whole-cell vaccination against MAP, increasing immune response and reducing bacterial load in tissues, and although some of the results presented here are in agreement with previous reports, these should be confirmed in a ruminant species. Mainly, because the differences observed in infection between both vaccination strategies could be in part influenced by the age of the animals and the period that the animals were under vaccination effects in each vaccination strategy (animals from the IVS group were vaccinated 2 months after animals of the VSI group meaning that in those 2 months the protective effect of vaccination could have decreased).

On the contrary to what may be traditionally believed, vaccination after infection has shown a slightly stronger protective effect than before infection. In any case, the protective mechanisms that operate behind MAP vaccination are unknown and further research is needed to understand why MAP infection progression and/or vaccination fail in some animals. In this case, rabbits can be a useful and non-expensive laboratory infection model to advance in the knowledge of MAP pathogenesis and vaccination.

## References

[1] Beard PM, Daniels MJ, Henderson D, Pirie A, Rudge K, Buxton D, Rhind S, Greig A, Hutchings MR, McKendrick I, Stevenson K, Sharp JM (2001). Paratuberculosis infection of nonruminant wildlife in Scotland. J Clin Microbiol.

[2] Pavlik I, Bartl J, Dvorska L, Svastova P, du Maine R, Machackova M, Yayo Ayele W, Horvathova A (2000). Epidemiology of paratuberculosis in wild ruminants studied by restriction fragment length polymorphism in the Czech Republic during the period 1995–1998. Vet Microbiol.

[3] Naser SA, Ghobrial G, Romero C, Valentine JF (2004). Culture of *Mycobacterium**avium* subspecies *paratuberculosis* from the blood of patients with Crohn’s disease. Lancet.

[4] Juste RA, Elguezabal N, Garrido JM, Pavon A, Geijo MV, Sevilla I, Cabriada J-L, Tejada A, García-Campos F, Casado R, Ochotorena I, Izeta A, Greenstein RJ (2008). On the prevalence of *M. avium* subspecies *paratuberculosis* DNA in the blood of healthy individuals and patients with inflammatory bowel disease. PLoS One.

[5] Bach H (2015). What role does *Mycobacterium**avium* subsp. *paratuberculosis* play in Crohn’s disease?. Curr Infect Dis Rep.

[6] Bastida F, Juste RA (2011). Paratuberculosis control: a review with a focus on vaccination. J Immune Based Ther Vaccines.

[7] Groenendaal H, Zagmutt FJ, Patton EA, Wells SJ (2015). Cost-benefit analysis of vaccination against *Mycobacterium**avium* ssp. *paratuberculosis* in dairy cattle, given its cross-reactivity with tuberculosis tests. J Dairy Sci.

[8] Wentink GH, Bongers JH, Zeeuwen AA, Jaartsveld FHJ (1994). Incidence of paratuberculosis after vaccination against *M. paratuberculosis* in two infected dairy herds. Zentralbl Veterinarmed B.

[9] Griffin JFT, Hughes AD, Liggett S, Farquhar PA, Mackintosh CG, Bakker D (2009). Efficacy of novel lipid-formulated whole bacterial cell vaccines against *Mycobacterium**avium* subsp. *paratuberculosis* in sheep. Vaccine.

[10] Juste RA, Alonso-Hearn M, Molina E, Geijo M, Vazquez P, Sevilla IA, Garrido JM (2009). Significant reduction in bacterial shedding and improvement in milk production in dairy farms after the use of a new inactivated paratuberculosis vaccine in a field trial. BMC Res Notes.

[11] Alonso-Hearn M, Molina E, Geijo M, Vazquez P, Sevilla IA, Garrido JM, Juste RA (2012). Immunization of adult dairy cattle with a new heat-killed vaccine is associated with longer productive life prior to cows being sent to slaughter with suspected paratuberculosis. J Dairy Sci.

[12] Garrido JM, Vazquez P, Molina E, Plazaola JM, Sevilla IA, Geijo MV, Alonso-Hearn M, Juste RA (2013). Paratuberculosis vaccination causes only limited cross-reactivity in the skin test for diagnosis of bovine tuberculosis. PLoS One.

[13] Fridriksdottir V (2000). Paratuberculosis in Iceland: epidemiology and control measures, past and present. Vet Microbiol.

[14] Kennedy D, Citer L (2010) Paratuberculosis Control Measures in Australia. In: Behr MA, Collins DM, CAB International RU (eds) Paratuberculosis: organism, disease, control. pp 330–343

[15] Saxegaard F, Fodstad F (1985). Control of paratuberculosis (Johne’s disease) in goats by vaccination. Vet Rec.

[16] Corpa JM, Pérez V, Sánchez MA, García Marín JF, Marín JF (2000). Control of paratuberculosis (Johne’s disease) in goats by vaccination of adult animals. Vet Rec.

[17] Reddacliff L, Eppleston J, Windsor P, Whittington R, Jones S (2006). Efficacy of a killed vaccine for the control of paratuberculosis in Australian sheep flocks. Vet Microbiol.

[18] Windsor PA, Eppleston J (2006). Lesions in sheep following administration of a vaccine of a Freund’s complete adjuvant nature used in the control of ovine paratuberculosis. N Z Vet J.

[19] Larsen AB, Hawkins WW, Merkal RS (1964). Experimental vaccination of sheep against Johne’s disease. Am J Vet Res.

[20] Windsor P (2006). Research into vaccination against ovine Johne’s disease in Australia. Small Rumin Res.

[21] Gwozdz JM, Thompson KG, Manktelow BW, Murray A, West DM (2000). Vaccination against paratuberculosis of lambs already infected experimentally with *Mycobacterium**avium* subspecies *paratuberculosis*. Aust Vet J.

[22] Gröschel MI, Prabowo SA, Cardona P-J, Stanford JL, van der Werf TS (2014). Therapeutic vaccines for tuberculosis: a systematic review. Vaccine.

[23] Beard PM, Rhind SM, Buxton D, Daniels MJ, Henderson D, Pirie A, Rudge K, Greig A, Hutchings MR, Stevenson K, Sharp JM (2001). Natural paratuberculosis infection in rabbits in Scotland. J Comp Pathol.

[24] Maio E, Carta T, Balseiro A, Sevilla IA, Romano A, Ortiz JA, Vieira-Pinto M, Garrido JM, de la Lastra JMP, Gortázar C (2011). Paratuberculosis in European wild rabbits from the Iberian Peninsula. Res Vet Sci.

[25] Greig A, Stevenson K, Perez V, Pirie AA, Grant JM, Sharp JM (1997). Paratuberculosis in wild rabbits (*Oryctolagus**cuniculus*). Vet Rec.

[26] Arrazuria R, Molina E, Mateo-Abad M, Arostegui I, Garrido JM, Juste RA, Elguezabal N (2015). Effect of various dietary regimens on oral challenge with *Mycobacterium**avium* subsp. *paratuberculosis* in a rabbit model. Res Vet Sci.

[27] Herrold RD (1931). Egg yolk agar medium for the growth of tubercle bacilli. J Infect Dis.

[28] Middlebrook G, Cohn ML (1958). Bacteriology of tuberculosis: laboratory methods. Am J Public Health Nations Health.

[29] Sevilla IA, Garrido JM, Molina E, Geijo MV, Elguezabal N, Vázquez P, Juste RA (2014). Development and evaluation of a novel multicopy-element-targeting triplex PCR for detection of *Mycobacterium**avium* subsp. *paratuberculosis* in feces. Appl Environ Microbiol.

[30] Towbin H, Staehelin T, Gordon J (1979). Electrophoretic transfer of proteins from polyacrylamide gels to nitrocellulose sheets: procedure and some applications. Proc Natl Acad Sci U S A.

[31] Mokresh AH, Czuprynski CJ, Butler DG (1989). A rabbit model for study of *Mycobacterium**paratuberculosis* infection. Infect Immun.

[32] Mokresh AH, Butler DG (1990). Granulomatous enteritis following oral inoculation of newborn rabbits with *Mycobacterium**paratuberculosis* of bovine origin. Can J Vet Res.

[33] Vaughan JA, Lenghaus C, Stewart DJ, Tizard ML, Michalski WP (2005). Development of a Johne’s disease infection model in laboratory rabbits following oral administration of *Mycobacterium**avium* subspecies *paratuberculosis*. Vet Microbiol.

[34] Kohler H, Gyra H, Zimmer K, Drager KG, Burkert B, Lemser B, Hausleithner D, Cussler K, Klawonn W, Hess RG (2001). Immune reactions in cattle after immunization with a *Mycobacterium**paratuberculosis* vaccine and implications for the diagnosis of *M*. *paratuberculosis* and *M*. *bovis* infections. J Vet Med B Infect Dis Vet Public Health.

[35] Muskens J, van Zijderveld F, Eger A, Bakker D (2002). Evaluation of the long-term immune response in cattle after vaccination against paratuberculosis in two Dutch dairy herds. Vet Microbiol.

[36] Stabel JR, Waters WR, Bannantine JP, Lyashchenko K (2011). Mediation of host immune responses after immunization of neonatal calves with a heat-killed *Mycobacterium**avium* subsp. *paratuberculosis* vaccine. Clin Vaccine Immunol.

[37] María Muñoz de Frutos (2014) Eficacia de una vacuna inactivada grente a la paratuberculosis bovina en un modelo experimental en terneros y su influencia en la patogenia de la enfermedad. PhD Thesis. Universidad de León

[38] Begg DJ, Griffin JFT (2005). Vaccination of sheep against *M*. *paratuberculosis*: immune parameters and protective efficacy. Vaccine.

[39] Corpa JM, Pérez V, García Marín JF (2000). Differences in the immune responses in lambs and kids vaccinated against paratuberculosis, according to the age of vaccination. Vet Microbiol.

[40] Hines ME, Turnquist SE, Ilha MRS, Rajeev S, Jones AL, Whittington L, Bannantine JP, Barletta RG, Gröhn YT, Katani R, Talaat AM, Li L, Kapur V (2014). Evaluation of novel oral vaccine candidates and validation of a caprine model of Johne’s disease. Front Cell Infect Microbiol.

[41] Ghosh P, Shippy DC, Talaat AM (2015). Superior protection elicited by live-attenuated vaccines in the murine model of paratuberculosis. Vaccine.

[42] Thomsen VT, Nielsen SS, Thakur A, Jungersen G (2012). Characterization of the long-term immune response to vaccination against *Mycobacterium**avium* subsp. *paratuberculosis* in Danish dairy cows. Vet Immunol Immunopathol.

[43] Nedrow AJ, Gavalchin J, Smith MC, Stehman SM, Maul JK, McDonough SP, Thonney ML (2007). Antibody and skin-test responses of sheep vaccinated against Johne’s Disease. Vet Immunol Immunopathol.

[44] Flynn JL (1993). An essential role for interferon gamma in resistance to *Mycobacterium**tuberculosis* infection. J Exp Med.

[45] Begg DJ, de Silva K, Carter N, Plain KM, Purdie A, Whittington RJ (2011). Does a Th1 over Th2 dominancy really exist in the early stages of *Mycobacterium**avium* subspecies *paratuberculosis* infections?. Immunobiology.

[46] Achkar JM, Casadevall A (2013). Antibody-mediated immunity against tuberculosis: implications for vaccine development. Cell Host Microbe.

[47] Balu S, Reljic R, Lewis MJ, Pleass RJ, McIntosh R, van Kooten C, van Egmond M, Challacombe S, Woof JM, Ivanyi J (2011). A novel human IgA monoclonal antibody protects against tuberculosis. J Immunol.

[48] Huda A, Jungersen G, Lind P (2004). Longitudinal study of interferon-gamma, serum antibody and milk antibody responses in cattle infected with *Mycobacterium**avium* subsp. *paratuberculosis*. Vet Microbiol.

[49] Dupont C, Thompson K, Heuer C, Gicquel B, Murray A (2005). Identification and characterization of an immunogenic 22 kDa exported protein of *Mycobacterium**avium* subspecies *paratuberculosis*. J Med Microbiol.

[50] Juste RA (2012). Slow infection control by vaccination: paratuberculosis. Vet Immunol Immunopathol.

[51] Bannantine JP, Everman JL, Rose SJ, Babrak L, Katani R, Barletta RG, Talaat AM, Gröhn YT, Chang Y-F, Kapur V, Bermudez LE (2014). Evaluation of eight live attenuated vaccine candidates for protection against challenge with virulent *Mycobacterium**avium* subspecies *paratuberculosis* in mice. Front Cell Infect Microbiol.

[52] Singh SV, Singh PK, Singh AV, Sohal JS, Sharma MC (2010). Therapeutic effects of a new “indigenous vaccine” developed using novel native “Indian bison type” genotype of *Mycobacterium**avium* subspecies *paratuberculosis* for the control of clinical Johne’s disease in naturally infected goatherds in India. Vet Med Int.

[53] Singh K, Chandel BS, Chauhan HC, Dadawala A, Singh SV, Singh PK (2013). Efficacy of ‘indigenous vaccine’ using native ‘Indian bison type’ genotype of *Mycobacterium**avium* subspecies *paratuberculosis* for the control of clinical Johne’s disease in an organized goat herd. Vet Res Commun.

[54] Santema W, Rutten V, Segers R, Poot J, Hensen S, Heesterbeek H, Koets A (2013). Postexposure subunit vaccination against chronic enteric mycobacterial infection in a natural host. Infect Immun.

[55] Berzofsky JA, Ahlers JD, Janik J, Morris J, Oh S, Terabe M, Belyakov IM (2004). Progress on new vaccine strategies against chronic viral infections. J Clin Invest.

